# Anxiety and Depression in Early Gestation and the Association with Subsequent Gestational Diabetes Mellitus in a Disadvantaged Population

**DOI:** 10.1007/s10995-023-03778-2

**Published:** 2023-10-12

**Authors:** Maleesa M. Pathirana, Prabha H. Andraweera, Shalem Leemaqz, Emily Aldridge, Margaret A. Arstall, Gustaaf A. Dekker, Claire T. Roberts

**Affiliations:** 1 Adelaide Medical School, The University of Adelaide, Adelaide Health and Medical Sciences Building, North Terrace, Adelaide, SA 5000 Australia; 2 Robinson Research Institute, The University of Adelaide, Adelaide, Australia; 3 Department of Cardiology, Lyell McEwin Hospital, Elizabeth Vale, Australia; 4 Flinders Health and Medical Research Institute, Flinders University, Bedford Park, SA 5042 Australia; 5 Division of Women’s Health, Lyell McEwin Hospital, Elizabeth Vale, Australia

**Keywords:** Gestational diabetes mellitus, Maternal health, Pregnancy

## Abstract

**Objectives:**

Evaluate the association between poor mental health and risk of developing gestational diabetes mellitus (GDM) in a cohort of women from a socioeconomically disadvantaged community.

**Methods:**

A total of 1363 nulliparous women with singleton pregnancies recruited to the Screening Tests to Predict Poor Outcomes of Pregnancy study in Adelaide, Australia. Women were assessed for mental health in the first trimester, including likelihood of depression, high functioning anxiety, perceived stress and risk of developing a mental health disorder. GDM was diagnosed based on the International Association of Diabetes in Pregnancy Study Group (IADPSG) criteria. Socioeconomic status was measured using the New Zealand Socioeconomic Index (NZSEI).

**Results:**

Complete mental health data was available for 1281 participants. There was no statistically significant difference in SEI, depression, risk of mental health issues, high functioning anxiety and perceived stress between women who developed GDM and those who did not. There was no difference in history of depression nor risk of developing a high mental health disorder in first trimester after adjusting for SEI, BMI in first trimester, smoking status in first trimester and maternal age between women with a GDM pregnancy and those who did not.

**Conclusions for Practice:**

There was no difference in markers of poor mental health in early pregnancy between women who subsequently did or did not develop GDM. Cohort participants were socioeconomically disadvantaged, potentially contributing to the lack of apparent differences in depression observed between groups. Socioeconomically disadvantaged women should be targeted in pre-conception planning to reduce risk of GDM.

## Significance

The literature on the association between common mental disorders and development of gestational diabetes mellitus (GDM) is inconsistent. Furthermore, this association has not been looked at in the context of a socioeconomically disadvantaged community.

What this study adds: There was no association seen between markers of poor mental health and GDM. The association between history of depression and development of GDM was attenuated by covariates such as SEI and maternal age. As the cohort is socioeconomically disadvantaged, it may be difficult to detect an association between poor mental health and development of GDM and therefore this indicates a need to explore this association further in similar cohorts, particularly as they are vulnerable to both poor physical health and psychological stress.

## Introduction

Gestational diabetes mellitus (GDM) is defined as hyperglycaemia in pregnancy, which is first diagnosed during pregnancy and affects 1 in 7 pregnancies in Australia (Andraweera et al., 2018; 2014). GDM poses a myriad of risks to both woman and child both during the perinatal period (macrosomia, birth injury, caesarean section, neonatal hypoglycaemia) and later in life when it is associated with poor metabolic and cardiovascular health (Andraweera et al., 2018; Pathirana et al., 2020a, b, 2021, 2022). Women with previous GDM are at a 7.5-fold increased risk of developing type 2 diabetes mellitus compared to those with no history of GDM (Bellamy et al., 2009). Conventional risk factors for GDM include, but are not limited to, family history, age and ethnicity (Sweeting et al., 2022). While these risk factors are not modifiable, other conventional risk factors such as obesity and hypertension are primary targets for GDM prevention strategies.

Common mental disorders (CMD) including anxiety and depression are significant maternal health problems. It has previously been shown that 7–20% of women in high-income countries experience antenatal depression, and 20–25% of women have an anxiety disorder during pregnancy (Hamel et al., 2019). Some studies have shown an association between antenatal depression and anxiety with development of gestational diabetes mellitus (Arafa & Dong, 2019; Atlaw et al., 2022; Beka et al., 2018; Thiele et al., 2022). Previous systematic reviews and meta-analyses have demonstrated a bi-directional association between type 2 diabetes mellitus and major depressive disorder (Nouwen et al., 2019; Yu et al., 2015) and it is thought that there is a similar association between antenatal depression, GDM and postpartum depression (Arafa & Dong, 2019). This association is thought to be instigated by hyperactivity of the hypothalamic–pituitary–adrenal axis (HPA), which causes an increase in circulating cortisol and insulin resistance (Moulton et al., 2015; Musselman et al., 1998). During pregnancy, there is already an elevation in cortisol and other inflammatory markers such as C-Reactive Protein and Interleukin-6, which is thought to promote insulin resistance that leads to development of GDM (Clark et al., 2019). However, some studies do not support an association between CMD and development of GDM (Katon et al., 2011; Wilson, Santorelli, et al., 2020a, 2020b). Furthermore, pertinent studies have not assessed this association against important covariates such as obesity and low socioeconomic status (SES), which contribute to both depression and the diabetic phenotype such as that seen in women with GDM (Schmitz et al., 2016). Socioeconomic status is important in the context of understanding the association between maternal depression and GDM. In Australia, SES significantly impacts burden of mental health disorders, and those who are disadvantaged often experience difficulty finding effective help for their mental health problems which may lead to poor physical health outcomes overall (Enticott et al., 2022). Women from regions of low socioeconomic status are more likely to experience complications of pregnancy, including GDM (Kim et al., 2018). Hence, understanding whether there is an association between depression and pregnancy complications may aid in improving access to clinical services for disadvantaged pregnant women.

Therefore, the aim of our study was to determine the association between markers of poor mental health and subsequent development of GDM in a cohort of pregnant women from a metropolitan socioeconomically disadvantaged community.

## Methods

### Study Population

The Screening Tests to predict poor Outcomes of Pregnancy (STOP) study was a prospective cohort study, where 1383 nulliparous women with singleton pregnancies were recruited from three major hospitals in Adelaide, Australia. Ethics approval was obtained from the Women’s and Children’s Hospital Human Research Ethics Committee (HREC/14/WCHN/90). This study was conducted as per the ethical standards of the Declaration of Helsinki. Women provided informed consent to participate in the study. Majority of the participants were recruited from the Lyell McEwin Hospital, which serves one of the lowest socioeconomic regions in urban Australia. Residents in this region experience some of the highest levels of chronic disease and mental illness across urban Australia (Torrens University, 2016). The primary objective of this study was to develop screening tests for the prediction of major pregnancy complications such as pre-eclampsia, spontaneous preterm birth, small for gestational age delivery and gestational diabetes mellitus*.* Women were excluded if they were at high risk for preeclampsia or delivering a small for gestational age infant or delivering preterm due to gynaecological history or underlying medical conditions (including known pre-existing chronic hypertension, being on hypertensive medication or having blood pressure > 160/100 mmHg at 15 weeks’ gestation) or if they had three or more miscarriages or terminations. Couples who received medical or surgical interventions that could modify pregnancy outcome were also excluded.

### Data Collection

Consenting pregnant women were recruited into the STOP study between 2015 and 2017. Research and clinical midwives collected information from women including demographics, smoking status, family, medical and gynaecological history. At the first antenatal visit (between 9 and 16 weeks’ gestation) anthropometric data including height, weight and waist circumference were collected. Socioeconomic status was ascertained using the New Zealand Socioeconomic Index (Whitaker et al., 1998), calculated based on the participant’s occupation, producing a score between 10 and 90, with a lower score reflecting greater disadvantage. Smoking status was classified as a binary variable both in the 3 months prior to pregnancy and in the first trimester (yes/no).

As part of routine clinical care, women completed the following questionnaires to ascertain their mental health status at the first trimester visit:

- Antenatal (psychosocial) Risk Questionnaire (ANRQ; Austin et al., 2013),
- Edinburgh Postnatal Depression Score (EPDS; Cox et al., 1987),
- Perceived Stress Questionnaire (PSS; Cohen et al., 1983),
- State and Trait Anxiety score-6 (STAI-6; Auerbach, 1973).

### Definition of Antenatal Mental Health Outcomes

Anxiety in pregnancy was defined using the STAI-6 score, where a score below 30 was defined as “low to no anxiety”, 31–49 “normal level of anxiety” and a score of 45–80 was defined as a participant having an “elevated state of anxiety”. Likelihood of depression was assessed using the EPDS, where “low risk” of depression was scored 0–9, “moderate risk” of depression in the following year score 10–12, and “likely depressed” score 13–30. Risk of perinatal mental health morbidity was assessed using the ANRQ, with a score > 22. Women were considered at high risk when answering yes to any of the following questions: 2A (Have you ever had 2 weeks or more where you felt particularly worried, miserable or depressed?), 2B (Do you have any other history of mental health problems?) 8 (Were you emotionally abused growing up?), 9 (Have you ever been sexually or physically abused?). Stress was assessed using the PSS, whereby a score between 0 and 13 was considered “low” stress, 14 and 26 “moderate” stress and 27 and 40 “high” perceived stress. History of depression was defined as variable coded ‘yes’ to the question “do you have a history of depression?”. Medication history was reported and data was analysed according to which participants were taking antidepressants.

### Diagnosis of GDM

GDM was diagnosed at 24–28 weeks of gestation according to the International Association of Diabetes in Pregnancy Study Group (IADPSG) criteria (i.e. one or more values equal to or exceeding: fasting plasma glucose of 5.1 mmol/L, and/or a 2 h plasma glucose level of 8.5 mmol/L following a 75 g Oral Glucose Tolerance Test (OGTT; Metzger et al., 2010). We also included women who, due to pre-existing risk factors, were diagnosed with GDM at 12 weeks’ gestation. This decision was made as descriptive analysis showed no difference in baseline parameters and mental health markers when excluding women diagnosed with GDM earlier than 24 weeks’ (data not shown).

### Statistical Analysis

Data were analysed using IBM SPSS Version 26. Univariate analyses were undertaken to assess women with GDM compared to women with non-GDM pregnancies for baseline variables, using χ^2^ test for categorical variables and *t*-test for continuous variables. Logistic regression analyses assessed the effect of having a history of depression (dichotomous grouping i.e. yes or no) on the risk of GDM, and having a high risk of having a mental health disorder (i.e. scoring high risk on the ANRQ) controlling for maternal age, BMI in first trimester, smoking status in first trimester and SEI, with data presented as odds ratio (95% CI). Variables were selected for logistic regression based on stepwise regression analysis and whether the variable was clinically associated with both development of CMD and GDM. Data are presented as Mean (SD) or N (%).

## Results

**Fig. 1:**
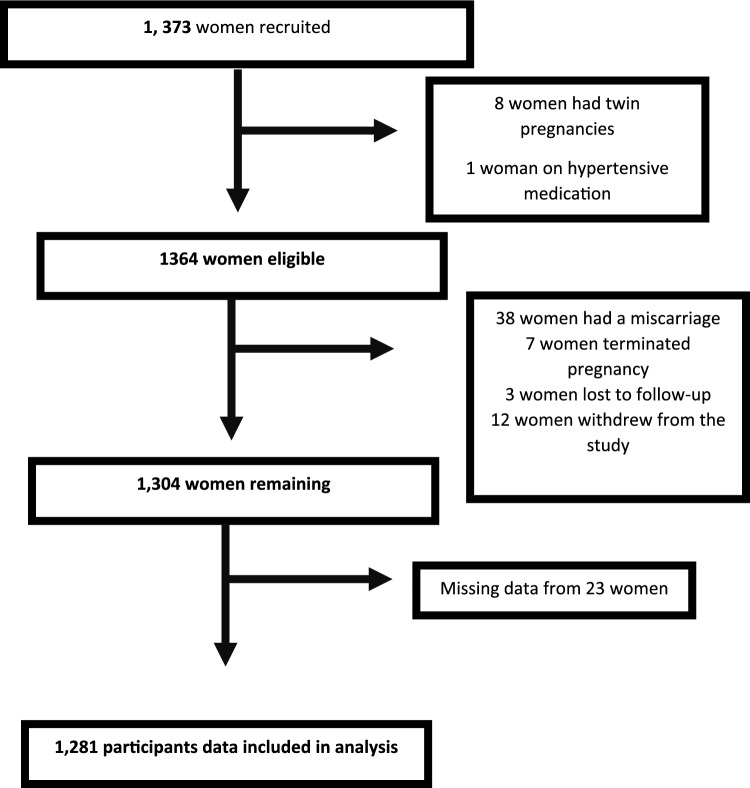
Flow chart of participants

The STOP study recruited 1383 pregnant women from 2015 to 2018. Some women were excluded from this analysis due to miscarriage, loss to follow-up, or twin pregnancy. Of those recruited, there are data available for 1300 with known pregnancy and birth outcomes. For this analysis, complete data for 1281 participants were available (Fig. 1). Of these, 196 women were diagnosed with GDM and 1085 experienced a non-GDM pregnancy. The participants in the latter group were women with an uncomplicated pregnancy and those who experienced pregnancy complications (but not including GDM) such as gestational hypertension, preeclampsia, spontaneous preterm birth and delivery of a small for gestational age (SGA) infant. Descriptive statistics are highlighted in Table 1. Caucasian ethnicity was lower in women diagnosed with GDM than those with a non-GDM pregnancy. Women with a pregnancy complicated by GDM were statistically significantly older compared to their non-GDM pregnant counterparts. Furthermore, BMI in first trimester was significantly higher in women later diagnosed with GDM compared to women with a non-GDM pregnancy [29.4 (7.3) vs. 27.5 (5.8), p = 0.002]. There was no significant difference in SEI nor smoking status between women diagnosed with GDM and those with a non-GDM pregnancy. The mean age, BMI and SEI score were similar for the cohort overall (Table 1). There were fewer women with GDM who delivered a SGA infant than those who did not develop GDM. There was no significant difference in history of depression nor use of antidepressants between women diagnosed with GDM compared to those with a non-GDM pregnancy. History of depression was reported in 27.3% of the whole cohort, and antidepressant use was recorded for 23.8% (Table 1).

**Table 1:** Characteristics of participants in early pregnancy

	Total	GDM (n = 196)	Non-GDM (n = 1085)	P-value
Caucasian ethnicity [N (%)]	1134 (82.5%)	140 (71.4)	920 (84.8)	–
Maternal age (Median years range)	26 (15 to 45)	27.9 (5.1)	25.6 (4.9)	**0.000**
Maternal Education [N (%)]				
Did not complete year 10	29 (2.1)	4 (2)	22 (2)	–
Year 10	261 (19)	27 (13.8)	219 (20)	
Year 12	306 (22.3)	42 (21.4)	248 (22.9)	
Certificate	488 (35.5)	74 (37.8)	384 (35.4)	
Bachelor	205 (15)	40 (20.4)	147 (13.5)	
Higher degree	80 (5.8)	9 (4.6)	61 (5.6)	
NZSEI* (Median and range)	29 (18 to 89)	29 (18 to 89)	29 (18 to 89)	0.500
BMI in first trimester (Median and range)	28 (15.8 to 61.4)	31.2 (17.8 to 59.4)	25.8 (15.8 to 61.4)	**0.002**
Other pregnancy complications (n =) (%)				
Preeclampsia	–	18 (9.2)	102 (9.4)	0.829
Gestational Hypertension		21 (10.8)	65 (6)	0.923
Small for gestational age		20 (10.2)	132 (12.2)	0.009
Maternal tobacco smoking [N (%)]				
Pre-pregnancy (3 months)	264 (20.6)	37 (18.9)	227 (20.9)	0.785
First trimester	216 (16.9)	34 (17.3)	182 (16.8)	0.523
History of depression [N (%)] Yes	375 (27.3)	60 (30.6)	286 (26.4)	0.217
History of antidepressant use for CMD [N (%)] Yes	249 (23.8)	43 (21.9)	184 (22.5)	0.867

*NZSEI a scale of 10–90 based on occupation. A lower score indicates increasing disadvantage

Bold indicates statistical significance of p < 0.05

Associations of mental health markers in early pregnancy between women later diagnosed with GDM and those with a non-GDM pregnancy are shown in Table 2. There was no difference seen between GDM and non-GDM groups for risk of developing a mental health disorder, likelihood of depression, perceived stress and state of anxiety.

**Table 2:** Association of mental health, likelihood of depression, stress perception and anxiety status in women with gestational diabetes in pregnancy compared to women with a non-GDM pregnancy

	Whole cohort N (%)	Gestational diabetes N (%)			Non-GDM N (%)			P-value
	High	Low	Moderate	High	Low	Moderate	High	
Risk of mental health disorder (ANRQ)^1^	581 (42.7)	101 (52.1)	–	93 (47.9)	626 (58.2)	–	450 (41.8)	0.113
Likelihood of depression (EPDS)^2^	106 (7.8)	168 (86.2)	13 (6.7)	14 (7.2)	895 (83.3)	93 (8.7)	87 (8.1)	0.572
Stress perception (PSS)^3^	41 (3.2)	93 (49.7)	88 (47.1)	6 (3.2)	568 (54)	453 (43)	32 (3)	0.567

		None-low anxiety	Normal level of anxiety	Elevated state of anxiety	None-low anxiety	Normal level of anxiety	Elevated state of anxiety	
Anxiety Status (STAI)^4^	149 (10.8)	99 (50.5)	65 (33.2)	28 (14.3)	558 (51.3)	389 (36)	111 (12.72)	0.165

^1^High risk of mental health disorder was based on an ANRQ score > 22 or answering yes to questions 2A, 2B, 8 or 9 (specified in methods)

^2^Low risk of depression was scored 0–9, moderate risk of depression was scored 10–12, likely to be depressed scored 13–30

^3^Low perceived stress was scored as 0–13, moderate perceived stress was scored 14–26 and high perceived stress was scored 27–40

^4^A score below 30 denotes “low to no anxiety”, 31–49 “normal level of anxiety” and 48–80 an “elevated state of anxiety”

We performed a logistic regression analysis to determine the association between having a history of depression, having a high risk of developing a mental health disorder in first trimester, or having an elevated state of anxiety in the first trimester and the risk of subsequent development of GDM, adjusting for SEI, BMI in first trimester, smoking status in first trimester, and maternal age. There was no significant association between having a history of depression and GDM after adjusting for covariates. Having a high risk for a mental health disorder in first trimester was not associated with GDM (Table 3).

**Table 3:** Association between history of depression and high risk of mental health disorder with risk of GDM based on logisitic regression

	Unadjusted	Adjusted^#^
History of depression	0.805 (0.577–1.123)	0.850 (0.596–1.212)
High risk of mental health disorder	0.781 (0.577–1.061)	0.772 (0.568–1.050)

^**#**^Adjusted for SEI, BMI in first trimester, smoking status in first trimester, and maternal age

## Discussion

In this cohort study in a socioeconomically disadvantaged population, we did not find a statistically significant association between parameters of women’s mental health during pregnancy and development of GDM. Furthermore, the prevalence of a history of depression, and that of being at high risk for mental health disorders, were not significantly different between women in the GDM and non-GDM groups.

Approximately 50% of women with GDM in pregnancy scored at high risk of developing a mental health disorder in their first trimester. This was also similar in the non-GDM group. The ANRQ assesses an individual’s psychosocial risk. A score of 23 or more is considered to be a clinically significant predictor of postpartum depression (Slavin et al., 2020). We sought to determine if there was an association between a high ANRQ score and risk of developing GDM. However, after adjusting for covariates such as age, BMI, smoking status and SEI there was no difference between groups.

Women from the STOP cohort were recruited from a community that is among the most severely disadvantaged in urban Australia (Liu et al., 2016). Mean SEI, as assessed on the basis of occupation, confirmed the high level of deprivation among many women in the cohort. Reports of psychological distress in the northern Adelaide region (i.e. a score of ≥ 10 or more on the K10 depression scale) are 20% higher than the national average, and mental health and behavioural problems are 5% higher than the national average (PHN, 2020). Women in this community predominantly have low levels of formal education, social support and income which all contribute to a higher risk of mental health disorders. Individuals with low social support and low SES have been shown to have a higher EPDS score, and higher rates of antepartum and postpartum depression than those who received adequate social support in a community of higher SES (Ahmed et al., 2019). The majority of the literature that has found an association between antenatal depression and risk of GDM assessed women from communities with an average or high SES [23–26]. This is likely due to the difficulty in engaging those from low SES populations in clinical research. However, a very pertinent study that assessed women from an area of severe disadvantage found that depression was not associated with GDM. Therefore, it is likely that any association between depression and subsequent GDM in a low SES community is masked due to the high risk of mental health disorders across all pregnant women in that community. Furthermore, associations between depression and GDM may be more confounded in our cohort because rates of obesity and other factors such as smoking, alcohol consumption, reduced exercise and diabetes are higher than the state and national averages (AIHW, 2019).

The northern Adelaide region experiences higher rates of domestic violence and other offences than other regions of Adelaide (AGO, 2016). The ANRQ assesses health history and social determinants of health such as physical, sexual and/or emotional abuse, and emotional and or practical support from a partner. It is quite likely that an association between mental health risk status and GDM could be masked in our cohort due to the high rate of poor social support seen in both GDM and non-GDM groups. Furthermore, psychosocial risk affects both physical health and diet, which would place these women at risk of obesity and development of diabetes (Gilbert et al., 2019).

Reports in the literature are inconsistent regarding the association between depression and subsequent GDM. Depression alters metabolism, specifically by elevating oxidative stress and cortisol which drive insulin resistance and elevations in blood glucose (Riggin, 2020). Similar to depression, anxiety and stress can promote increased HPA activity, thereby promoting higher cortisol and arginine vasopressin secretion which subsequently impact insulin levels in the body and promote insulin resistance (Mishra et al., 2020). Some studies suggest an association, while others do not. Hinkle et al*.* assessed depression scores based on the EPDS in first trimester and found that depression in early pregnancy was associated with a 2-fold increased risk of developing GDM after adjusting for relevant covariates (Hinkle et al., 2016). A similar association was seen by Atlaw et al*.*, showing that antenatal depression as scored by EPDS in early pregnancy was associated with a 4-fold increased risk of GDM even after adjustment for covariates (Atlaw et al., 2022). Wilson et al*.* found no evidence of an association between common mental disorders in the pre-natal period and GDM, for which depression and anxiety were diagnosed based on ICD diagnostic scoring (Wilson, Santorelli, et al., 2020a, 2020b). However, Byrn et al. (2015) showed a significant association between depression/mood disorder and subsequent GDM in multivariate analyses (Byrn & Penckofer, 2015). A very recent meta-analysis also showed that GDM is associated with depressive symptoms. However, the analysis was highly heterogeneous due to variation of how depression and anxiety were diagnosed (Wilson, Newham, et al., 2020). In our study, depression was self-reported and not clinically assessed. Therefore, the severity of depression between participants may vary. Other studies assessed depression in different ways including retrospective data linkage and EPDS (Beka et al., 2018; Bowers et al., 2013; Byrn & Penckofer, 2015; Thiele et al., 2022). It may be important to consider severity of depression for future studies, as this may influence the severity of maternal metabolic dysfunction and insulin resistance and thereby influence glucose tolerance in pregnancy.

There is still discrepancy in the literature regarding the association between anxiety and GDM. Our study showed that high functioning anxiety was more common, but not statistically significant, in women with GDM compared to non-GDM. Mishra et al*.*, found a significant association between high-perceived stress and GDM (Mishra et al., 2020) while Silveria et al. showed no association between perceived stress during early or mid-pregnancy and subsequent GDM (Silveira et al., 2014). Lee et al*.* in a cross-sectional study in Malaysia found that 40% of women who had GDM reported anxiety symptoms as per the Depression and Anxiety Stress Scale (DASS), and 10% of women reported stress (Lee et al., 2019).

However, these studies did not assess the correlation between perceived stress and diagnostic OGTT glucose levels. Therefore, it may be important to examine glycaemic levels and perceived stress, particularly as the HAPO study has shown that glucose levels below conventional diagnostic criteria at the time for GDM were associated with poor antenatal maternal and neonatal outcomes (Metzger et al., 2008).

The strengths of our study include the large cohort with 15% of women with GDM, which is comparable to the national average of approximately 15% of pregnant women (AIHW, 2019). Our study also captures one of the lowest socioeconomic urban regions of Australia, where chronic diseases such as type 2 diabetes and cardiovascular disease are highly prevalent. We assessed many risk factors, such as stress perception, anxiety and risk of common mental disorders. Our limitations include not having a clinical diagnosis of depression or anxiety. In our cohort, nearly half of the participants were considered at high risk of developing a mental health disorder at their antenatal booking visit. However, the ANRQ does not look at current symptomology and provides a holistic concept of antenatal maternal health, therefore it is best interpreted adjunctly with the EPDS or a prior clinical diagnosis of depression. Furthermore, the prevalence of antenatal depression is significantly higher in disadvantaged communities. In this cohort, we report low social support, lower education status and psychological factors such as stigma attached to mental health disorders that impact maternal mental health.

Another limitation of this study is that we were unable to do a power calculation a priori, therefore the results that we have found should be interpreted with caution, however we believe that this study provides a basis to further investigate this association in adequately powered studies of cohorts with socioeconomic disadvantage.

Furthermore, as this population is very disadvantaged (median SEI score of 29) it may be difficult to detect differences between GDM and non-GDM participants regarding mental health outcomes. Our population was primarily Caucasian. Therefore, our results may not be generalizable to women of other ethnicities.

## Conclusion

We did not find a significant difference between women with GDM in pregnancy and women with a non-GDM pregnancy for history of depression and markers of depression, anxiety and stress in early pregnancy. This may be due in part to the low SES in our cohort. Therefore, patients of socioeconomic disadvantage who are likely to already be experiencing poor mental health should be assessed in the pre-conception and early pregnancy period to reduce the risk of developing pregnancy complications. Future research should aim to assess risk of GDM in women with clinically diagnosed depression and assess different levels of obesity and socioeconomic disadvantage to explore these associations further.
